# Developing the CARE intervention to enhance ethical self-efficacy in dementia care through the use of literary texts

**DOI:** 10.1186/s12910-023-00926-9

**Published:** 2023-06-29

**Authors:** Sigurd Lauridsen, Frederik Schou-Juul, Anna Paldam Folker, Peter Simonsen, Marie-Elisabeth Phil, Sofie Smedegaard Skov

**Affiliations:** 1 The National Institute of Public Health, University of Southern Denmark, Copenhagen, Denmark; 2 Department Department of Language, Culture, History and Communication, University of Southern Denmark, Odense, Denmark

**Keywords:** Dementia care, Ethical intervention, Self-efficacy

## Abstract

**Background:**

Dementia care is essential to promote the well-being of patients but remains a difficult task prone to ethical issues. These issues include questions like whether manipulating a person with dementia is ethically permissible if it promotes her best interest or how to engage with a person who is unwilling to recognize that she has dementia. To help people living with dementia and their carers manage ethical issues in dementia care, we developed the CARE intervention. This is an intervention focused on promoting the ethical self-efficacy of people living with dementia and carers, i.e., their confidence that they can manage ethical issues when they occur. The purpose of this paper is to explain and discuss how we have developed the CARE intervention to promote the ethical self-efficacy of people living with dementia, their family, and professional carers through a specific and, we believe, new use of literary texts.

**Methods:**

The CARE intervention has been developed in two phases: First, we conducted a needs assessment of the occurrence of ethical issues in dementia care and the need for an intervention to support people living with dementia and their carers in managing such issues. Second, in a design phase, we developed the CARE intervention to meet identified needs.

**Results:**

To address identified ethical issues in dementia care we designed the CARE intervention as a workshop format where people living with dementia and carers can meet, discuss literary texts, and deliberate on how to solve such issues. The workshop is structured by the following elements: An agenda of ethical issues, a collection of literary cases exemplifying ethical issues, a moderator with an understanding of dementia care, and an overview of the ethical principles relevant to the discussion of ethical issues. >This workshop concept is operationalized in three applications tailored to meet the specific ethical issues of each of the study´s three target groups: people living with dementia and family carers, professional and family carers, and professional carers.

**Conclusion:**

We conclude the paper by stating that it is possible to develop an intervention that promotes the ethical self-efficacy of people living with dementia and family and professional carers.

**Supplementary Information:**

The online version contains supplementary material available at 10.1186/s12910-023-00926-9.

## Background

Still more people suffer from dementia [1]. In 2015, 47 million people worldwide have dementia, and the number is predicted to increase to 75 million in 2030 and 132 million by 2050 [2]. At the same time there are no effective treatments of dementia. Alzheimer´s disease, for instance, is the most prevalent form of dementia (accounting for up to 50% of cases) and while four drugs are currently licenced for its treatment none of them provides any long-lasting effect [3].

This creates a care challenge to people living with dementia and their carers because while dementia care is essential to promote the well-being of people living with dementia and their carers it is also difficult and prone with ethical issues [4, 5].

Ethical issues in dementia care can e.g. be a conflict between respecting the self-determination of a person with dementia, in terms of what she wants, and acting in what from a given professional´s point of view is in her best interest [6]. Other ethical issues concern how caregivers can prioritize justly among the needs of people with dementia they care for or, in some situations, how to balance avoiding causing harm to people with dementia with allowing them to make their own decisions. In this paper, we primarily define ethical issues according to the ethical framework of principlism [4]. Principlism states that four moral principles, i.e., beneficence (promoting people´s best interest), non-maleficence (avoid causing people harm), respect for autonomy (supporting people´s self-determination), and justice (fairly distributing benefits, goods, and risks) [7], constitute *prima facie* moral norms that should be followed and that ethical issues occur when they conflict. We also incorporate elements from the ethical literature on dignity in dementia care [8], on how to balance e.g. dignity and self-determination, and from feminist literature on relational autonomy concerning how the autonomy of people with dementia can be enhanced by carers [9].

To solve the care challenge described above much attention has been given to answer the ethical issues in dementia in the theoretical ethical literature on the subject [10–13]. For instance, The British Nuffield Council on Bioethics in 2009 published a report on dementia ethics in response to the increasing prevalence of dementia in the UK. The report concluded, among many things, that carers of people living with dementia needed more support and access to appropriate education to better tackle the ethical problems they meet on a daily basis than is currently provided and presented an ethical framework aimed at helping people living with dementia and their carers [5]. In 2009 an EU network on ethics in dementia was also created and operated by Alzheimer Europe though it was discontinued after some years [14]. Recently, a new COST Action EU network, Ethics in Dementia, focused on working with ethical issues in dementia care, has been established [15].

Notwithstanding, considerably less attention has been devoted to involving people living with dementia and their carers in devising tools that can help them manage ethical issues in living with dementia [16], although studies show the importance of having such tools and involving stakeholders in making them [17, 18]. This is, probably, partly because the academic field of ethical issues in dementia care to a high degree has focused on advance directives and -planning concerning end-of-life decisions and less on everyday ethical issues [19, 20] and partly because ethicists in general tend to believe that ethical issues are best solved theoretically without the involvement of the people who meet the issues in practice [21].

To help remedy this problem – that people living with dementia and their carers have not been sufficiently involved in making tools to manage ethical issues in dementia care – we have developed the CARE intervention in close collaboration with people living with dementia and professional and family carers: an intervention developed to support people living with dementia and carers to manage ethical issues in dementia care. More specifically, the goal of the intervention is to promote the *ethical self-efficacy* of people living with dementia and their carers, i.e., their confidence in their own ability to manage ethical issues when they occur when living with dementia. While the concept of self-efficacy, i.e. a person’s belief that she has the capability to act in ways necessary to reach specific goals [22], has previously been used as an outcome measure in studies in other domains of dementia care [23], [24], it has not been applied specifically to ethical decision-making in dementia care. The assertion is that when individuals with dementia and their caregivers have improved ethical self-efficacy, they feel more confident in their ability to navigate and master the ethical challenges that may arise throughout the course of the illness.

The research aim of this paper is to describe the development of the CARE intervention, which enhances ethical self-efficacy among people with dementia, their families, and professional carers, by utilizing literary texts in a novel manner.

Methods.

The CARE intervention has been developed by an interdisciplinary research group with participants from the University of Southern Denmark and Rudersdal Municipality, comprising: a research manager with a background in philosophy and public health who had extensive experience in working with ethical issues in dementia care; a PhD student with a background in public health science; a PhD student with a background in philosophy; a post doc with a background in literature studies and a municipal coordinator with a background in public health science.

**Fig. 1:**
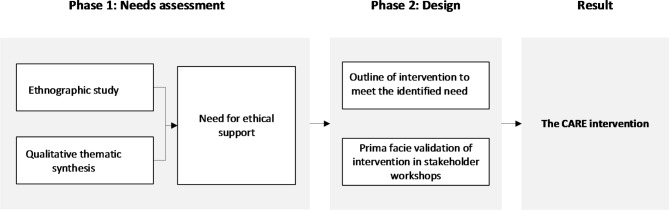
Design process

It has been developed in two phases: First, we conducted a needs assessment that investigated the occurrence of ethical issues in dementia care and the need for an intervention to support people living with dementia and their carers in managing such issues. Second, in a design phase, we developed the CARE intervention to meet identified needs (see Fig. 1).

### Phase 1: needs assessment

In this phase we explored what ethical issues and what support carers and people living with dementia have: first, in an ethnographic study we investigated how ethical issues emerge among carers and people living with dementia in two nursing homes and one day care center in Rudersdal municipality. Second, we conducted a qualitative thematic synthesis of guidelines distributed to people living with dementia and their family carers in Denmark.

#### An ethnographic study

To explore how ethical issues emerge in dementia care and are perceived by people living with dementia and their carers we conducted an ethnographic study of dementia care at three different care facilities in the municipality of Rudersdal: an activity centre, a somatic nursing home and a nursing home specialised in dementia. Somatic nursing homes in Denmark provide care for citizens with significant and lasting physical and/or psychosocial disabilities, and therefore also accommodate citizens with dementia. The three institutions were selected in collaboration with the municipality with the aim of reflecting the institutional variations in services for citizens with dementia at different stages of their disease.

The study comprised observations and interviews with people living with dementia and family and professional carers focusing on generating insights into which and how different ethical issues appeared in these groups, which, taken together represent a full dementia trajectory. Data was collected in November – December 2020 and again in February 2021. The PhD student who conducted the fieldwork was a part of the daily life at the institutions for three weeks at each location. This involved, for example, participating in meals, spending time and interacting with residents and staff members in common areas, and participating in formal and informal activities.

The study comprised a total of 39 observations lasting from 1.5 to 8 h, as well as the following interviews: four interviews with people with early-stage dementia, eight interviews with family carers, and five focus group interviews with professional carers (n = 17). Interviews were conducted using a semi-structured interview guide informed by ethical literature on dementia [4]. All interviews were subsequently transcribed *ad verbatim*. Observations were conducted using an observation guide informed by ethical literature on dementia [4]. We observed everyday situations at the nursing homes (e.g., meals, formal and informal activities) with special interest in the interactions between and among residents and caregivers. Behaviours, situations that seemed challenging, fragments of conservations, and descriptions of physical surroundings were written down as field notes.

All data from the observations were imported to the qualitative analysis software programme NVivo 12 and coded using thematic analysis [25]. The analyses of the ethnographic study showed that carers and people living with dementia face a number of ethical issues in their everyday lives, including how to talk to a person about opening up about their illness to those around them, if the person does not want to, and how to respect self-determination and privacy etc. The full design and results of this study are forthcoming in a separate publication.

#### A qualitative thematic synthesis

To broaden and further qualify the knowledge of the occurrence and variety of ethical issues when living with dementia, we also completed a qualitative thematic synthesis of guidelines to people living with dementia and family carers. The aim was to clarify what (if any) guidance people with newly diagnosed dementia and their family carers receive to manage ethical issues, and what needs remain unmet. Data were collected from January to March 2021.

The synthesis included 653 references; 555 remained after duplicates were removed. Data were collected via online search engines and manual contact with relevant governmental, municipal and patient organisations from November 2020 to January 2021. They wereselected based on formal criteria, such as publication year, target group and public availability. The data were controlled for content of ethical issues and recommendations. After screening for formal inclusion criteria, 59 references remained, and applying qualitative control for content of ethical issues and recommendations, it ultimately resulted in the selection of a sample of 15 guidelines. Data was imported to NVivo 12 and analysed with content analysis. The analyses identified four specific subcategories of prevalent ethical issues: being concerned with issues arising in conjunction with disclosure of private or sensitive matters; vulnerability and acceptance of dependence; changes in what is perceived as dignified behaviour; and decision-making and autonomy. The results of this study have been published in *Dementia* [16].

### Phase 2: design

The design phase of the CARE intervention was conducted in two separate stages: first, we outlined an intervention to address the need for ethical support identified in the needs assessment. Second, we *prima facie* validated and refined this intervention in workshops with people living with dementia, professional and family carers.

#### Outlining the intervention

The ethical issues we uncovered in our needs assessment were, to a large extent, shared by various target groups in dementia care, including people living with dementia, professional and family carers [9]. Hence, we decided that the intervention should be directed at all three target groups and the setting of the intervention should be day centres, frequented by people with dementia living at home, and nursing homes that house people with late moderate and severe dementia (see Table 1 for an overview of which target groups were addressed in which arena).

**Table 1:** Target groups and arenas

	Target groups	Arenas
1	People living with dementia living at home and their family carers	Day Center
2	Family and professional carers	Nursing Home
3	Professional carers	Nursing Home

Because ethical issues occur when moral principles conflict, we decided that the intervention should be designed as a workshop format where people living with dementia and carers could meet and deliberate on how to solve such conflicts. Also, we decided that the ethical issues to deliberate on should be presented as concrete cases rather than as abstract problems. While we considered different types of interventions, e.g. online or written communication material to people living with dementia and their carers, we decided that it was important to actively involve them face-to-face in deliberations on how to manage ethical issues, thus fostering peer-interaction and cooperative problem solving [26, 27].

To facilitate a thorough discussion of the ethical issues, we decided to present them through literary cases that express actual life situations [28]. Drawing on the tradition of narrative medicine, we hypothesised that using literary texts would be a useful way of fostering improved understanding of mutual experiences and values in dementia care [29]. To identify literary cases that exemplify the identified ethical issues, we conducted an extensive literary study of 15 Danish and Anglophone works of contemporary prose, poetry, autobiography, and fiction dealing with dementia. 10 works were autobiographical, and five were fictional. Criteria for selecting these works included works written from various viewpoints to gain insight into life with dementia from the perspective of care workers, people living with dementia and their next of kin, and works that poignantly describe life with dementia and directly engage without necessarily providing solutions to ethical issues in relation to dementia care. The most important criterium for selecting a literary work was that it should express a relevant ethical issue (see Table 2) in a setting and manner we considered close to Danish dementia care practice. After reading through the books, we selected six works in which we identified text excerpts for use in the intervention (see Table 3 for a list of the books we have used).

**Table 2:** List of books from which we have used text excerpts

Title	Examples of scenarios from the texts	Author	Type	Publica-tion year
No man’s land*	An elderly man who is frail and delirious expresses a desire to die at a nursing home. However, given his lack of autonomy, should caregivers fulfill his request?	Kirsten Thorup	Fiction	2004
Thawed snow and forsythia*	The author describes how they informed those around them about their wife’s dementia and the various reactions they received, highlighting the importance of discussing how to communicate about dementia.	Thomas Bredsdorff	Autobio-graphical	2017
Somebody I used to know	A woman with dementia describes how she, along with her daughters, is about to fill out a power of attorney for the future and shares her thoughts on not wanting her children to look after her in the future. Therefore, she delegates the decision regarding transition to a nursing home to her daughters	Wendy Mitchell	Autobio-graphical	2018
Gratitude	An elderly woman with cognitive frailty hides a bottle of whisky in her nursing home room, raising the question of privacy in long-term care facilities.	Delphine de Vigan	Fiction	2019
Dementia, dilemmas and star moments*	An adult child narrates her mother’s transition to a nursing home, including how her father one day reaches a breaking point regarding keeping the mother at home. She shares her conflicting emotions and thoughts about her own role. Have I done everything I could?	Lone Carmel and Annette Thuesen	Autobio-graphical	2020
I still see you*	A relative describes how she gradually takes over her husband’s responsibilities, such as planning their son’s celebration and writing emails for him along sharing her thoughts about wanting to continue to involve her husband in decisions as long as it remains meaningful.	Julie Rubow	Autobio-graphical	2020

*Danish book titles translated by us

**Table 3:** The nine ethical issues the CARE intervention address

	Target Groups		
	People living with dementia living at home and family carers	Family and professional carers	Professional carers
**Ethical** **issues**	How to engage with a person who is unwilling to recognize that he or she has dementia	How to coordinate care responsibilities between family and professional carers	How to prioritize the needs among residents at nursing homes
	This may involve a conflict between respecting the self-determination of the person with dementia and promoting her best interest	This may involve a conflict between respecting the autonomy of the family carer and the autonomy of the professional carer	This may involve a conflict between promoting the best interest of the person with dementia and allocating resources justly
	How to communicate with surroundings, e.g. friends and relatives, about having dementia if the person with dementia fears stigmatization	How to respect the privacy of people living with dementia when family and professional carers communicate	How to balance the need to sometimes use manipulation in the care of a person with dementia with respecting his or her dignity
	This may involve a conflict between respecting the self-determination of the person with dementia and promoting her best interest	This may involve a conflict between respecting the self-determination of the person with dementia and promoting her best interest	This may involve a conflict between respecting the self-determination of the person with dementia and promoting her best interest
	How to talk about future ethical issues, like future power of attorney, transition to nursing home etc., likely to come	How for family and professional carers to balance the needs of the person with dementia with the needs of other residents at the nursing home	How to respect the privacy of residents at nursing homes while at the same time providing optimal care
	This may involve a conflict between the principles of not doing harm (non-maleficence) and promoting the best interest of the person with dementia	This may involve a conflict between promoting the best interest of the person with dementia and allocating resources justly	This may involve a conflict between respecting the self-determination of the person with dementia and promoting her best interest.

#### ***Prima facie*** validation

Subsequently, this outline of the intervention was validated in three workshops with professional and family carers and people living with dementia.

The first workshop was held in September 2020 and was with professional carers (n = 10), including two nurses, five healthcare workers and three blue-collar workers, all working at nursing homes caring for people with dementia. The aim of the workshop was to test the relevance of the issues identified in the ethnographic study and, if necessary, to adjust them and to test whether literary cases describing ethical issues in dementia would be perceived as a suitable method for conversing on these issues. The professional carers were all experienced in working with people living with dementia. Some had backgrounds as nurses and social and health assistants while others were assistants with no educational background in the field of health. They went from having a few years to more than ten years of experience working with people living with dementia. The workshop lasted two hours. Four researchers and a coordinator from Rudersdal municipality participated in the workshop.

The second workshop was also completed in September 2020 and was held with family carers (n = 12). The aim of the workshop was to investigate whether the ethical issues earlier identified in the needs assessment were relevant to this target group. The participants´ experience with dementia spanned from an adult child whose mother had recently died from dementia to a woman whose husband had only recently been diagnosed with Alzheimer´s disease. Three researchers and a coordinator from Rudersdal Municipality participated in the workshop. One of the researchers facilitated the workshop while the two others continuously took notes. The workshop lasted two hours.

The last workshop was with people with early-stage dementia (n = 6). It was completed in January 2021. The aim of the workshop was to investigate the relevance of developing an intervention aimed at supporting management of ethical issues in dementia and whether the use of literary cases presented a suitable method for conversing on these issues. The workshop was conducted at a day centre for people living with dementia. In addition to the six persons with dementia a professional carer from Rudersdal Municipality also participated in the workshop. Two researchers conducted the workshop. The workshop lasted 45 min.

## Results

In this section we present the generic design of the CARE intervention, the ethical issues it addresses and its operationalization in three specific applications that address the individual needs of the project´s three target groups.

### The CARE intervention

To meet the need identified in our needs assessment, we designed the CARE intervention as a workshop concept where people living with dementia and carers meet and have a facilitated deliberation on ethical issues in dementia care. The design of the workshop concept was structured by the following elements:

- An agenda of ethical issues.
- A collection of literary cases exemplifying relevant ethical issues.
- A moderator with an understanding of dementia care and familiarity with the methodology of the intervention.
- An overview of the ethical principles and considerations relevant to the discussion of ethical issues to enable the moderator to facilitate the workshop.

The agenda constitutes the composition of the workshop and, notably, the ethical issues to be discussed among workshop participants.

To facilitate a discussion of these issues, as already mentioned, the CARE intervention makes use of literary cases. The idea was that to have a rich but sensitive discussion of the typically very complex ethical challenges faced by people living with dementia and carers, the issues should not be discussed as abstract ethical difficulties, nor as intimately tied to the real-life situations of people involved, but rather as concrete problems embedded in life situations as they are presented in literary texts. Thus, we explicitly wanted to utilise one of the main strengths of literary texts, which is that they are not solely bound by the factual, but also make use of the imagination to invent fictions that may stimulate new thinking and insight. This is especially true for difficult and tabooed subjects that lack predetermined outcomes, except the outcome of making the participant even more aware of the dilemma at hand [30, 31]. Interesting similar work has already been conducted using literature in connection with dementia care [28].

In the workshops we held for people living with dementia, professional and family carers when designing and validating the intervention, everyone endorsed the idea of using literary texts to discuss ethical issues, but especially the professional carers had strong opinions on the character of these cases. It was very important to them that they were realistic and accurately reflected their everyday practice. To accommodate their comments several text cases were replaced. The workshop with people living with dementia importantly confirmed that discussion of ethical issues by the use of literary cases was seen as appropriate for this target group.

The CARE intervention is to be facilitated by a moderator. The idea is that a moderator reads a literary excerpt aloud (which will also be handed out) and then asks participants open questions, such as ‘What is the dilemma at play here?’ or ‘Do you think this action was appropriate?’ The participants then consider the text excerpts and questions individually, in couples or in groups, depending on the specific application of the intervention. Through this use of literature as a medium to frame a discussion of ethical issues, we have aimed to create spaces for dialogue where people living with dementia and carers can meet and recognise themselves in literary texts that represent ethical issues and thereby constructively reflect upon and work towards personal solutions to the challenges they face in their respective life situations. Because narrative literary texts, as Martha Nussbaum influentially suggests, have “the power to make us see the lives of the different with more than a casual tourist’s interest” (p. 88) even as they do not provide concrete solutions to ethical issues that literary characters have, but instead engage the reader in an open-ended search for solutions typically modelled on their own situation, we hypothesised that these texts were especially good at enabling readers to find their own best solutions [32].

Finally, it is a requirement that the workshop, when people living with dementia are involved, is led by a moderator with extensive knowledge of and experience with dementia care, e.g. a dementia coordinator or a dementia nurse.

### Ethical issues

The CARE intervention concept outlined so far only describes the generic features of the intervention. To address the needs of the project´s specific target groups the overall intervention concept had to be operationalized. Each target group – people living with dementia and their family carers, family and professional carers and professional carers – faces an individual set of ethical issues which the intervention must address.

In total, we uncovered nine ethical issues in our needs assessment. Three per target group. All issues refer to situations where the four principles of principlism, beneficence (best interest), non-maleficence (not doing harm), respect for autonomy and justice, can conflict and thus leave people living with dementia, family, and professional carers in uncertainty on how to act. Although the ethical issues identified within the auspice of principlism correspond closely to the ethical issues we identified in the needs assessment, respect for privacy and dignity were also identified in this assessment as values championed by professional and family carers.

The list of the nine ethical issues is not intended to be exhaustive. To ensure that the issues that are dealt with at the CARE workshops reflect the needs of the participants, their relevance will continuously be assessed, and they will be replaced if necessary. See Table 2 for an overview of the nine ethical issues.

### Three applications of the CARE intervention

**Fig. 2:**
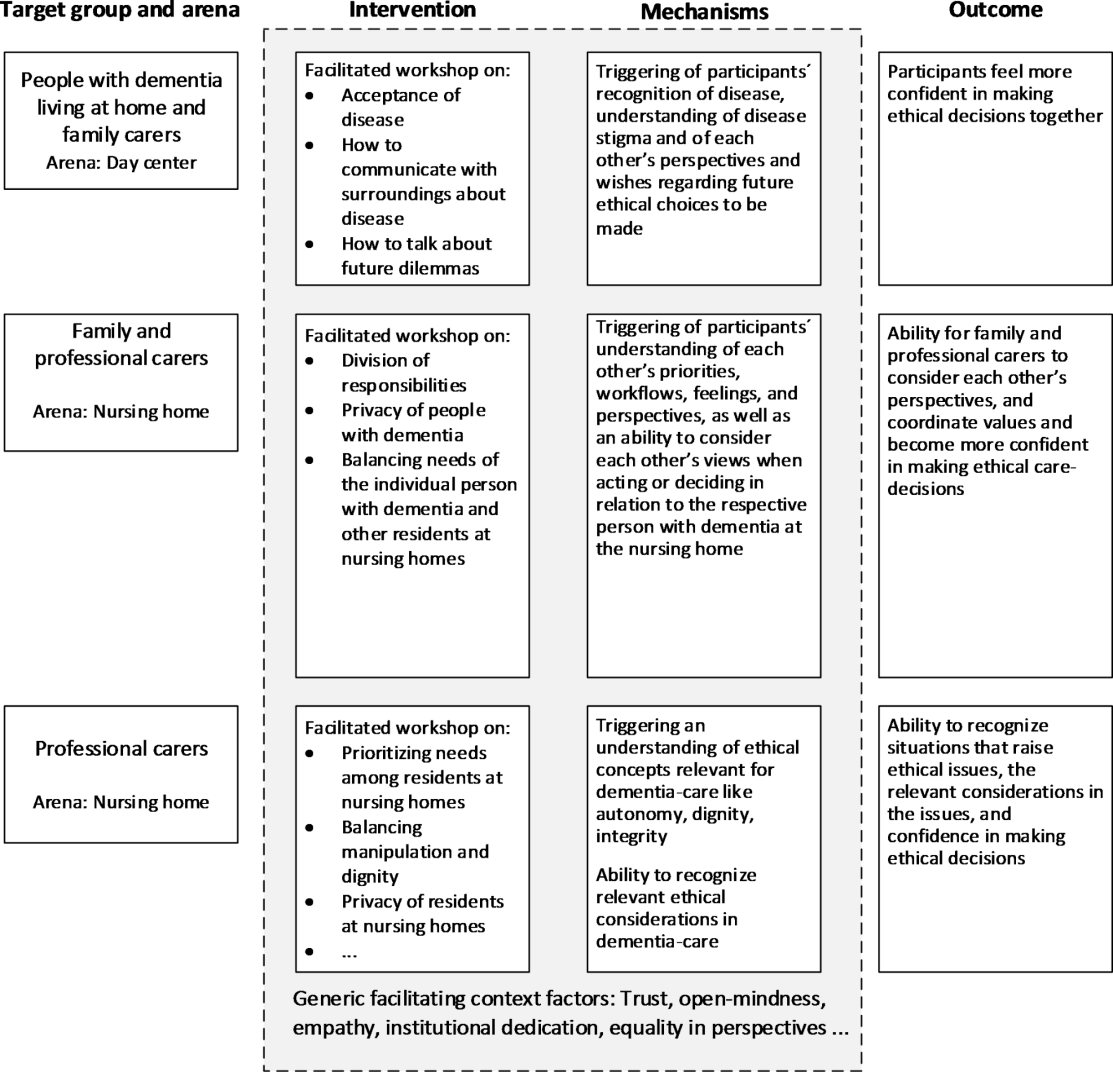
Program theories for the three specific applications

To address these nine ethical issues, we operationalized the CARE intervention in three independent specific applications, each containing a workshop agenda consisting of the ethical issues relevant to the target group in question, a collection of literary excerpts exemplifying the issues, an overview of the ethical principles and considerations at play in the issues and a guide on how to moderate a discussion of the issues using the collections of literary texts and ethical principles. Subsequently, we have designed a programme theory for each application, explaining how we expect the specific interventions to lead to improved self-efficacy regarding managing ethical issues for the respective target groups (see Fig. 2).

### First application: family carers and people living with dementia

The aim of the first application of the CARE intervention was to initiate deliberation among people living with dementia and their family carers on topics such as what it means to have a dementia disease, how to communicate with family, friends, and other surroundings about the disease and how to prepare for future decisions concerning issues such as transition to a nursing home. The hypothesis was that by having a conversation based on the literature on these topics with both their relatives and other people, persons living with dementia and their carers would obtain a better awareness or intelligence of their emotions and more confidence in managing ethical issues. Research on emotional intelligence, self-efficacy, and nursing has shown that there is a correlation between emotional intelligence, self-efficacy, and self-esteem among certain groups, such as nurses and individuals with intestinal stoma. These studies suggest that emotional awareness and intelligence can enhance confidence in some individuals. Given this evidence, it is possible that emotional intelligence could also benefit people with dementia and their caregivers. Thus, we hypothesize that improving emotional awareness and intelligence could boost the confidence or self-efficacy of individuals with dementia and their caregivers [33, 34]. The workshop was planned to last two hours.

### Second application: family and professional carers

The next application of the CARE intervention focuses on the collaboration between family and professional carers in caring for a person with severe dementia in a nursing home setting. We know from our ethnographic studies and workshops with professional and family carers that difficulties of collaboration between these two groups may arise due to, among other things, differences in values and relations to and perceptions of the person with dementia. Our hypothesis was that engaging in one another’s perspectives through literary cases and conversations on issues in dementia care would improve the collaboration between family carers and health professionals and make them more confident in making ethical care decisions. The workshop was planned to last two hours.

### Third application: professional carers

The third application focused exclusively on health professionals working in nursing homes. The purpose was to provide them with the tools required to identify, analyse, and act in relation to ethical issues in dementia care. Our hypothesis was that the moral sensitivity of these professionals would be enhanced by introducing them to ethical principles and using literary cases that describe ethical issues from the perspective of health professionals, family carers and even people living with dementia. This would improve their understanding of ethical issues and their confidence in making decisions concerning these types of issues. The implementation of this intervention was planned as two connected workshops, each lasting four hours.

## Discussion

People living with dementia and their carers face ethical issues every day, ranging from questions such as how to engage with a person with dementia who is unwilling to accept that she or he has the disease or how to decide whether manipulating a person with dementia is ethically permissible if it promotes her best interest. These issues have, for the most part, been given serious attention in the bioethical literature, and theoretical answers have been suggested [5, 35]. Little research, however, has focused on developing interventions to make the issues more manageable for those affected by them [36–38]. In this paper, we have explained how we, through collaboration with people living with dementia, carers and other stakeholders, have developed an intervention that fills this gap.

The CARE intervention has important affinities with similar ethics interventions that stress the importance of ethical reflection though developed for different settings and target groups [39–41]. Some ethicists would disagree with this approach to ethical issues and argue that the task is to apply theoretical solutions to the ethical issues in dementia rather than to empower people living with dementia and their family and professional carers to make up their own answers [42]. For instance, it has been argued that questions of resuscitation and life-prolonging treatment have been answered through ethical theory and have been successfully institutionalised as advance directives [43, 44]. To others, however, the idea of advance directives is flawed [20, 45]. Thus, some degree of disagreement exists for most, if not all, ethical issues in dementia care. This does not imply that ethical theory is unimportant in answering issues in dementia care. However, if people affected by dementia are to embrace principles and possible solutions developed in the field of bioethics, they need to find them meaningful and valuable, as well as be able to imagine them as applicable to their own situations [46]. Theoretical solutions to ethical issues may qualify and enrich a dialogue on how to manage the issues, but these solutions most often cannot substitute for an ongoing open-ended dialogue among those affected by dementia-causing diseases [47]. To address ethical issues in dementia care, we have adopted a practice-based approach that leverages the perspectives and experiences of those who face these challenges on a daily basis. Our approach draws on ethical theory in terms of the ethical framework of principlism, which helps identify ethical issues in dementia care. At the same time, we assert that ethical theory alone is insufficient for addressing the complexities of dementia care. Therefore, we engage people with dementia and their caregivers in facilitated workshops to collaboratively identify and develop solutions that work for their unique situations. By involving those directly impacted by the ethical issues, identified by the framework of principlism, we ensure that the solutions are relevant and practical, and reflect the values and preferences of the people affected. Ultimately, this approach, we assume, results in more meaningful ethical decision-making in dementia care.

The CARE intervention may also be criticised for being overly naïve. The change we pursue is that people affected by dementia can become more confident in managing ethical issues after scrutinising them in a deliberative process. This may seem like a plausible theory when it comes to family and professional carers, but less so when it comes to people living with dementia. The reason for this is that the theory of change only works if people receiving the intervention can recognise ethical issues, balance considerations relevant to the issues and make decisions on how to address them. These capabilities are the kind of competences lost by people living with dementia; therefore, developing an intervention may appear overly naïve. It seems to involve a group of people who, because of their disease, are prevented from reaching the outcome the intervention is designed to produce.

For two reasons, though, we do not believe that this criticism applies. Firstly, timing is important. People living with dementia can still be involved in ethical discussions and can still improve their ethical self-efficacy in the early phase of the disease [27, 48]. Involving people living with dementia in decision-making care enables an informed and respectful conversation with, for example, their spouses or children regarding which decisions to make when the disease progresses and counteracts the alienation that people living with dementia tend to experience as a consequence of their disease [49]. Secondly, though people living with dementia eventually will forget what they have talked about at the workshop, a longer lasting effect may occur for family carers having shared these conversations in the early stage of the disease. Sensitive topics that are difficult to address in everyday life may be taken up, such as future transition to a care facility, and having had a conversation on these topics may make them confident that they can make the right ethical decisions later when such choices must be made [50].

### Strengths and weaknesses

It is, we believe, a strength of the CARE program that the basis of the intervention, and the needs of target groups, have been thoroughly researched in both a comprehensive ethnographic study and a qualitative thematic analysis of guidelines to people living with dementia and their family carers. It is also a strength that stakeholders have been strongly involved in both outlining its design and in identifying the ethical dilemmas it addresses. It may be a weakness that the developed intervention includes three applications and hence is somewhat complex. The reason for this was that all the project’s stakeholders reported a great need for tools to be developed that would help them deal with ethical issues specific to their context.

### Further research

It is an important next step to evaluate the feasibility of the CARE intervention. Such a study will include an evaluation of whether people living with dementia and carers experience the intervention to be valuable and one that leads to improved ethical self-efficacy and whether it is practically possible to implement it in a municipal context etc. [51]. An assessment of the feasibility of the intervention will then allow a decision on the appropriateness of evaluating it further in a realistic evaluation design [52] or a cluster-randomised controlled trial to obtain the highest level of evidence of the effect of the intervention [53]. Also, more work needs to be done on investigating the relation between ethical self-efficacy and reasonable ethical decision-making.

## Conclusion

In this paper, we have presented and discussed the development process of the CARE intervention, which aims to support family and professional carers as well as people living with dementia in dealing with ethical issues related to dementia care. To achieve this goal, we employed a deliberation-based approach that draws on literary texts.

Planners of intervention programmes who work with ethical issues in dementia care, or who address similar sensitive and complex issues, can benefit from our experience in using participatory techniques, in the form of workshops, to engage key stakeholders and vulnerable groups in intervention development. The developed CARE intervention and its model for discussing complex and delicate matters by use of literary cases may also be helpful for researchers and planners who work more generally on engaging vulnerable groups in the deliberation of sensitive topics. Future studies will uncover the CARE intervention´s feasibility and eventually its effect on ethical self-efficacy among family and professional carers and people living with dementia.

## Electronic supplementary material

Below is the link to the electronic supplementary material.

Additional File 1: Evaluating Needs for Implementation of the CARE Intervention.

## Data Availability

Data from the qualitative thematic analysis of guidelines to people living with dementia and their relatives consist of Danish texts and research notes. Data from the workshops conducted to design the CARE intervention comprise research notes in Danish. The data that support the findings of this study are available from Sigurd Lauridsen but restrictions apply to the availability of these data, which were used under license for the current study, and are not publicly available. Data are available from the authors upon reasonable request, and with permission of Sigurd Lauridsen. Interview guides translated into English can be found here a supplementary file.
