# Supplementary material for: Developing the CARE intervention to enhance ethical self-efficacy in dementia care through the use of literary texts
Source: BMC Med Ethics. 2023 Jun 29;24:45. doi: 10.1186/s12910-023-00926-9 (PMC10311821; doi:10.1186/s12910-023-00926-9)
Supplement: Supplementary file 1 — Additional File 1: Evaluating Needs for Implementation of the CARE Intervention. [file 12910_2023_926_MOESM1_ESM.docx]

Evaluating Needs for Implementation of the CARE Intervention

Interview and observation guides

1. Interview with citizens with dementia

- Introduction of the interviewer (and possibly an observer)
- Distribution of participant information and introduction to the research aim:

*The purpose of this project is to gain insight into the difficult situations and challenges you experience in your everyday life. This insight will serve as the basis for the development of an intervention that can support you and your family. To understand what it's like to live with dementia, we believe in asking and listening to those who are directly affected by the condition. That's why I'm conducting this interview with you.*

- In this interview, I will ask you about yourself and your daily life, including the challenges you face in relation to living with dementia. We will also discuss what brings you joy in your daily life and how your environment can best support you.
- I would like to record the interview on a voice recorder if that is okay with you. What you share with me will be treated and stored confidentially. Only the group of researchers working on the project will have access to the interview. We might use some quotes from the interview for later articles, but your statements will remain completely anonymous.
- I expect the interview to last approximately 45 minutes to one hour.
- I would like to record this interview if it is okay with all of you? What you share will be treated confidentially. We may use some quotes from the interview in later articles, but your statements will remain completely anonymous.
- Please write your names on these slips of paper and place them in front of you.
- Do you have any questions before we begin?

Presentation

I would like to start by asking you to tell me a little about yourself, including:

- Your name and age
- Do you live alone or with someone?
- What type of dementia do you have?
- When were you diagnosed with the disease?

## Daily life

1. Can you tell me a bit about what your daily life looks like?
   - What activities do you enjoy?
   - What is important for you to spend time on?
   - Are there things you wish you had more of in your daily life?
2. How does a good day and less good day look like for you?
   - How does a less good day affect you?
   - How do you cope with challenging days?

## Ethical challenges

1. How has your daily life changed since being diagnosed with dementia?
   - Do you feel that others treat you differently? In what way?
2. What do you find difficult or challenging?
   - In which situations do these difficulties arise?
   - How do you handle those challenging situations?
   - Can you talk to anyone about these challenges? If not, how do you imagine someone could support you?
   - What or who can help you deal with challenging situations? Family members and/or healthcare professionals?

## Relationships and support

1. What is you experience of being associated with the day center?
   - What aspects are good or less good?
   - How do you perceive your relationship with the staff and peers?
2. Do you feel that you can be yourself?
   - What is necessary for you to feel like you can be yourself?
3. How can your family best support you?
   - What obstacles may prevent your family from supporting you?
4. How can healthcare professionals best support you?
   - What obstacles may prevent healthcare professionals from supporting you?

I would like to ask you a difficult question. If it becomes too personal and you don't feel like answering, that's perfectly fine.

1. If there comes a day when you can no longer make good decisions for yourself, how would you like your surroundings to help you?
   - How should they make dignified decisions on your behalf?
   - Do you trust that others can make dignified decisions for you? Why or why not?
2. If you were to imagine something that would be undignified for you, what would it be?

## Conclusion

1. Is there anything you would like to add at the end or any topics we haven't covered yet?
2. Lastly, I would like to hear how it has been for you to participate in this interview?

Thank you very much for taking the time to participate in the interview.

I wish you all the best in the future.

1. Interview with family caregivers

- Introduction of the interviewer (and possibly an observer)
- Distribution of participant information and introduction to the research aim:

*The purpose of the project is to gain insight into ethical dilemmas faced by individuals living with dementia, their relatives, and healthcare professionals. This insight will form the basis for developing an intervenion that can hopefully help support some of the difficult situations that you may experience.*

- In this interview, I will ask about your perceived challenges in relation to your mother/father/spouse and the healthcare professionals closely involved in their daily life. I will also ask about how your surroundings can best support you, including any services and initiatives you feel are lacking in managing the challenges you face.
- I would like to record the interview on a voice recorder if that is okay with you. What you share with me will be treated and stored confidentially. Only the group of researchers working on the project will have access to the interview. We might use some quotes from the interview for later articles, but your statements will remain completely anonymous.
- I expect the interview to last approximately 45 minutes to one hour.
- I would like to record this interview if it is okay with all of you? What you share will be treated confidentially. We may use some quotes from the interview in later articles, but your statements will remain completely anonymous.
- Do you have any questions before we begin?

Presentation

I would like you to start by telling us a bit about yourself, including:

- Your name and age
- What do you do on a daily basis?
- Who is your family composed of?

## Daily life

1. Could you briefly tell us the story of your spouse/parent, including when he/she received their dementia diagnosis?
   - What diagnosis has he/she been given?
   - Did you notice any changes before the diagnosis was made?
2. How did you experience the transition to a nursing home?
   - Who helped you handle it?
   - Did you miss any forms of support during this transition?
   - How did your spouse/parent react to the transition?

## Ethical challenges and support

1. What do you find difficult or challenging in your relationship with your spouse/parent?
   - Why is it difficult?
   - How often does it occur?
   - What considerations/needs conflict with each other, as you see it?
2. How do you handle challenging situations?
   - Can you talk to someone about the challenges? Who?
   - If not, how do you imagine someone could support you?
   - What or who can help you handle challenging situations?
3. If you think about the whole process/journey that you and your spouse/parent have been on since dementia entered your life, what kind of services/initiatives do you feel are lacking for you as a relative?
   - Do you think there are differences in the needs of relatives depending on whether they are (adult) children or spouses of a person with dementia?
   - How do they differ?

## Relationships and collaboration with healthcare professionals

1. How is your relationship with the staff at the nursing home?
   - What works well or less well in the collaboration concerning your mother/father/spouse?
2. How can the healthcare professionals at the nursing home best support you?
   - What might hinder healthcare professionals from supporting you?

I would like to ask you a difficult question. If it becomes too personal and you don't feel like answering, that's perfectly fine.

1. If there comes a day when your spouse/parent can no longer make good decisions for themselves, how would you like decisions regarding them to be made?
   - How do you make dignified decisions for your spouse/parent?
   - Do you trust the staff who are close to your spouse/parent on a daily basis to make dignified decisions for him/her? Why or why not?
2. If you were to imagine something that would be undignified for him/her - what would it be?

## Conclusion

1. Is there anything you would like to add at the end or any topic we may have missed?
2. Lastly, I would like to hear how it has been for you to participate in the interview?

Thank you very much for taking the time to participate in the interview.

I wish you all the best in the future.

1. Focus Group Interview with Healthcare Professionals

- Introduction of the interviewer and observer
- Distribution of participant information and introduction to the research project’s aim:

*The purpose of the project is to gain insight into the ethical dilemmas faced by people with dementia, their relatives, and healthcare professionals. This insight will serve as the basis for the development of an intervention that can hopefully help alleviate some of the difficult situations that one may experience.*

- The purpose of this group interview is to gain insight into your experiences working with citizens with dementia and their relatives. All viewpoints are welcome, so there are no "right" or "wrong" things to say in this setting. The interview will be conducted as a group interview, which means that I will ask some questions that you are welcome to discuss with each other. I encourage you to share your immediate thoughts and experiences, even if you have a different perspective from your colleague.
- The expected duration of the interview is 1-1.5 hours.
- I would like to record this interview if it is okay with all of you? What you share will be treated confidentially. We may use some quotes from the interview in later articles, but your statements will remain completely anonymous.
- Please write your names on these slips of paper and place them in front of you.
- Do you have any questions before we begin?

Presentation

I would like each of you to start by telling a little bit about yourselves, including:

- Name and age
- Educational background
- Job title

## Roles and relationships

To begin with, I would like you to describe how our work life looks like.

1. Can you start by telling me about what your daily life looks like?
   - What contributes to making a workday good?
   - How does a less good workday look like?
2. Please describe your role in relation to the people with dementia you work with.
   - What is your primary task?
   - What do you spend the most time on?
   - What is your relationship like?
3. Please describe your role in relation to the relatives you interact with.
   - What is your primary task?
   - What do you spend the most time on?
   - What are your relationships like with the relatives?

## Ethical dilemmas

As we have already discussed, a significant part of your work is based on relationships. Engaging in relationships with different individuals and navigating various considerations simultaneously is not always straightforward. As you know, we are particularly interested in the challenging situations that can arise when working with people with dementia. Therefore, I would like you to reflect on…

1. What situations do you find difficult or challenging in your daily lives?
   - When do these situations arise?
   - Why do you think they arise?
2. How do you handle challenging situations?
   - What skills do you rely on when dealing with a difficult situation?
   - Do you draw on education, experience, or gut feeling, for example?
   - Do you involve anyone in managing it? Who? And how?
3. Can you imagine what (or who) could help you handle challenging situations?
   - In what way would it/they be able to help you?

## Conclusion

1. Is there anything you would like to add at the end or any topics we haven't covered yet?

Thank you very much for participating in this interview. I wish you all the best in the future.

1. Guide for participant observations

*Register and observe in as much detail and neutrality as possible - avoid translations, interpretations, or evaluations of statements and expressions.*

| **Facts** | |
| --- | --- |
| Date  Location and context *Formal/informal*  Who is present? *Citizens/residents, relatives, staff* |  |
| Descriptive notes (avoid interpretations) | |
| Descriptions of the location (physical space, furniture, objects, layout, smell, noise, etc.)  Activities/events/tasks *What are the participants doing?*  *Formal and informal activities*  Interactions  *Who interacts with each other?*  *How do they interact?*  *Reactions to the interactions?* Dialogues *Who is conversing with whom?*  *What are they talking about?*  *Reactions to the conversations?* |  |
| **Analytical aspects** |  |
| Value foundation* *Visible values? Invisible or implicit values?*  *Rules? Attitudes? Norms?*  Ethical questions and dilemmas  *What is discussed/perceived as challenging?*  *For whom is it challenging?*  *When and why do the challenges arise?*  *Which values and norms are challenged?*  *Reactions to the challenges?*  Handling  *How are the challenges handled?*  *Reactions to the handling?*  Unspoken practices and taken-for-granted assumptions.  *Habits? Traditions?*  Norm or rule violations (situations that appear awkward/deviating) *Reactions to them? Expressed vs. unexpressed (e.g., through body language)* |  |
| **Methodological notes** | |
| Access negotiations *(permissions/rejections)*  Researcher's presence *(positive/negative influences)* |  |

*Value concepts that people assess and discuss ethics on can include (according to Jacob Birkler):

- Respect/disrespect
- Responsibility/irresponsibility
- Professionalism/unprofessionalism
- Openness/closedness
- Dignity/indignity
- Engagement/disengagement
- Autonomy/heteronomy
